# Retrieving Historical Cases of Aujeszky’s Disease in Sicily (Italy): Report of a Natural Outbreak Affecting Sheep, Goats, Dogs, Cats and Foxes and Considerations on Critical Issues and Perspectives in Light of the Recent EU Regulation 429/2016

**DOI:** 10.3390/pathogens10101301

**Published:** 2021-10-09

**Authors:** Vincenzo Di Marco Lo Presti, Ana Moreno, Anna Castelli, Dorotea Ippolito, Antonino Aliberti, Benedetta Amato, Maria Vitale, Michele Fiasconaro, Flavia Pruiti Ciarello

**Affiliations:** 1 Istituto Zooprofilattico Sperimentale “A. Mirri” della Sicilia, Via G. Marinuzzi 3, 90129 Palermo, Italy; vincenzo.dimarco@izssicilia.it (V.D.M.L.P.); antoninoaliberti.vet@gmail.com (A.A.); benedetta_am@yahoo.it (B.A.); maria.vitale@izssicilia.it (M.V.); michele.fiasconaro@izssicilia.it (M.F.); pruitiflavia@outlook.it (F.P.C.); 2 National Reference Centre for Aujeszky Disease, Istituto Zooprofilattico Sperimentale della Lombardia e dell’ Emilia Romagna “B. Umbertini”, Via Bianchi 9, 25124 Brescia, Italy; anamaria.morenomartin@izsler.it (A.M.); anna.castelli@izsler.it (A.C.)

**Keywords:** pseudorabies, multi-species, SuAHV-1

## Abstract

Aujeszky’s disease is caused by *Suid alphaherpesvirus 1*, and its main reservoir host is the pig. However, other species are also susceptible. Infection with this virus causes a severe neurological clinical picture named Aujeszky’s disease, usually accompanied by itching and death a few days after the onset of symptoms. This study reports a multi-species outbreak of Aujeszky’s disease that occurred in Sicily, which led to the death of 2 goats, 15 sheep, 2 dogs, 2 cats and 2 foxes. The diagnosis was made by culture, indirect immunofluorescence on brain samples and confirmed by biological test on rabbits. This study reports the first cases of Aujeszky’s disease in Italy in cats, goat and sheep. The finding of Aujeszky’s disease in several species in Sicily suggests a potential epizootic risk. In such areas where a multi-host system is recognised, an analysis of the risk factors should be carried out in order to develop targeted strategies for the control and eradication of the disease. The critical issues that hinder the control of Aujeszky’s disease in the studied territory and perspectives for eradication in the light of EU regulation 429/2016 are also discussed.

## 1. Introduction

Aujeszky’s disease (AD), also called pseudorabies, is a notifiable disease caused by *Suid alphaherpesvirus 1* (SuAHV-1) that is an alphaherpesvirus member of the *Herpesviridae* family, genus *Varicellovirus* [1]. The pig and wild boar (*genus* *Sus)* are considered the principal reservoir hosts [2,3,4,5]. Notoriously SuAHV-1 does not have a species-specific tropism, and these animals serve as a source of infection for ruminants, monogastric herbivores, carnivores and rodents [6,7,8]. Indeed, the causative agent of AD was firstly isolated from the brain of a bovine, a dog and a cat, by the veterinary pathologist and microbiologist Aladár Aujeszky in Hungary in 1908 [9]. Furthermore, past and recent reports of AD in humans underline its potential zoonotic role and its public health implications [10,11,12,13]. Since the 1950s, many cases of AD have been reported worldwide in species other than pigs and wild boar [14], all epidemiologically related to direct or indirect contacts with pigs/wild boar infected with a wild type of SuAHV-1. In Italy, only sporadic cases of AD have been reported in non-natural host, such as foxes [15,16], dogs [2,3], wolves [17], bears [18] and cattle [19]. Infection in non-natural hosts generally occurs by faecal-oral or aerosol routes but, given the high stability of SuAHV-1in the environment, indirect infection by exposure to infected fomites is also described [20]. Additionally, exposure to the modified live vaccines developed for pigs may cause disease in sheep [21], dogs [22,23] and foxes [16]. AD in non-natural hosts is sporadic, usually with a lethal outcome. The clinical symptoms are related to the neurotropic nature of the SuAHV-1 that, after an initial replication phase in peripheral neurons, spreads centripetally to the central nervous system (CNS). The incubation period varies from three to six days. The animals might die suddenly without premonitory signs or might develop a typically neuropathic pruritus [14], and/or non-specific symptoms (high temperature, discomfort, continuous bellowing, whirling around, convulsions, opisthotonos), followed by death within few days. AD diagnosis in animals other than pigs is often based on clinical signs (incoercible itching and/or nervous symptoms) and possible contact with pigs or wild boar, as reported in the clinical history. Although serology serves as a useful screening tool in pigs, it is not commonly used in other species, as most of the animals die before detectable serum antibodies are produced [24,25]. Given the recent reports of AD in cattle in the Sicilian multi-host environment, endemic for the disease [19], the authors retrospectively analysed the clinical cases of AD in species other than the pigs in this territory. The present work reports a singular outbreak of AD in both domestic (sheep, goat, dogs and cats) and wild species (foxes), that occurred in Sicily in December 1996, shortly before the application of the first mandatory national plan for the control and eradication of AD in Italy (DM. 11/04/1997). Furthermore, to the best of our knowledge, the present work reports the first ever cases of AD in sheep, goats and cats in Italy. The detection of AD in several non-natural hosts in Sicily, both past and recent [19], suggests a potential epizootic risk. Despite the efforts used to eradicate the disease, it remains persistent and prevalent in Sicily, especially in particular protected areas where a multi-host epidemiological system is recognized [19]. Furthermore, we consider it appropriate to discuss some of the critical issues that have hindered the control of AD in Sicily. 

## 2. Results

### 2.1. Anamnesis, Epidemiological Investigation and Clinical Examination

**Figure 1:**
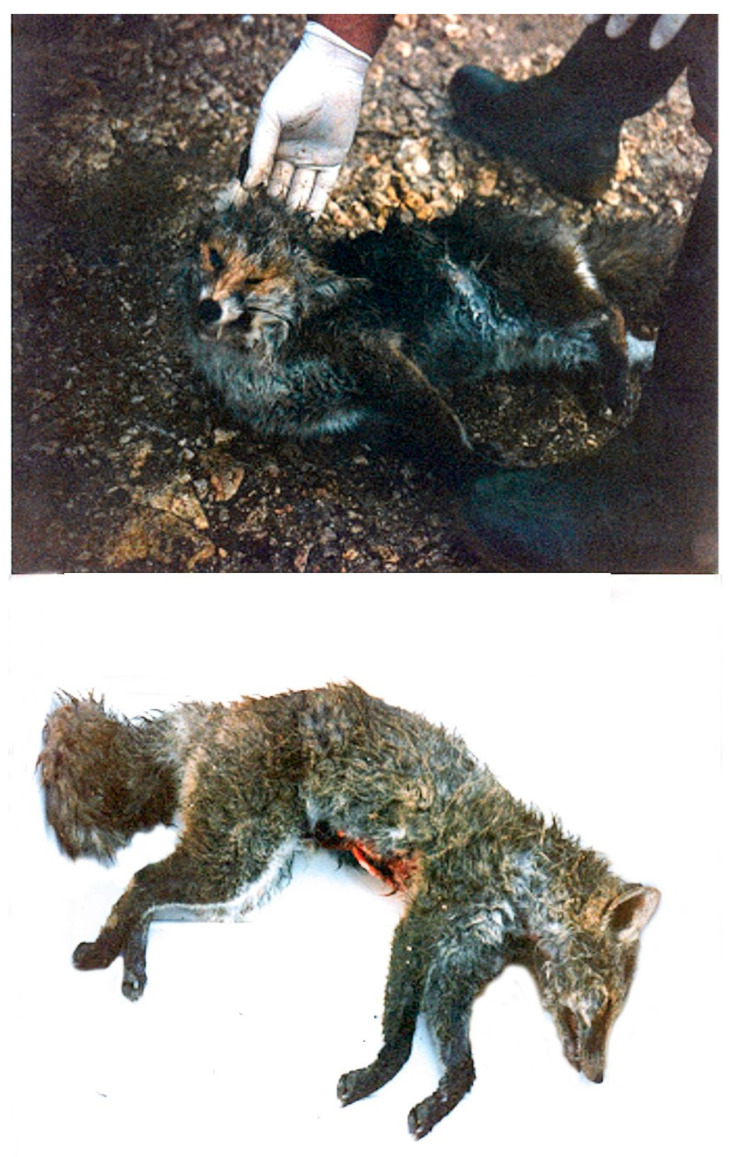
A fox affected by Aujeszky’s disease, found dead near the farm where the outbreak was located.

**Figure 2:**
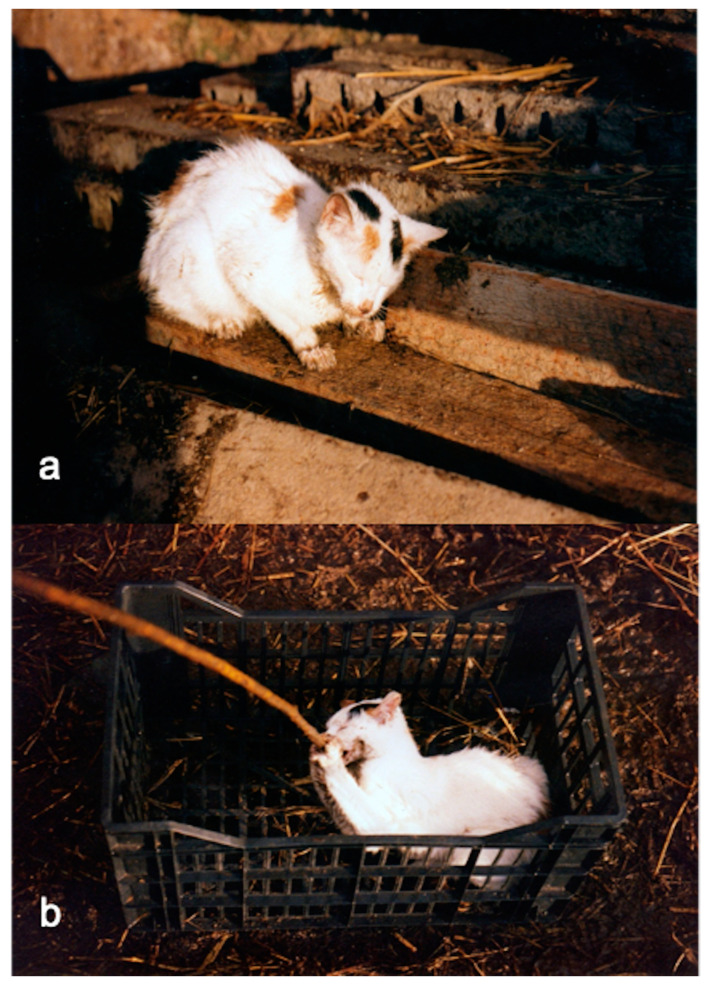
A cat with symptoms of AD. (**a**) scratching lesions of the labial region and face; (**b**) aggressive attitude and excessive response to external stimuli.

**Figure 3:**
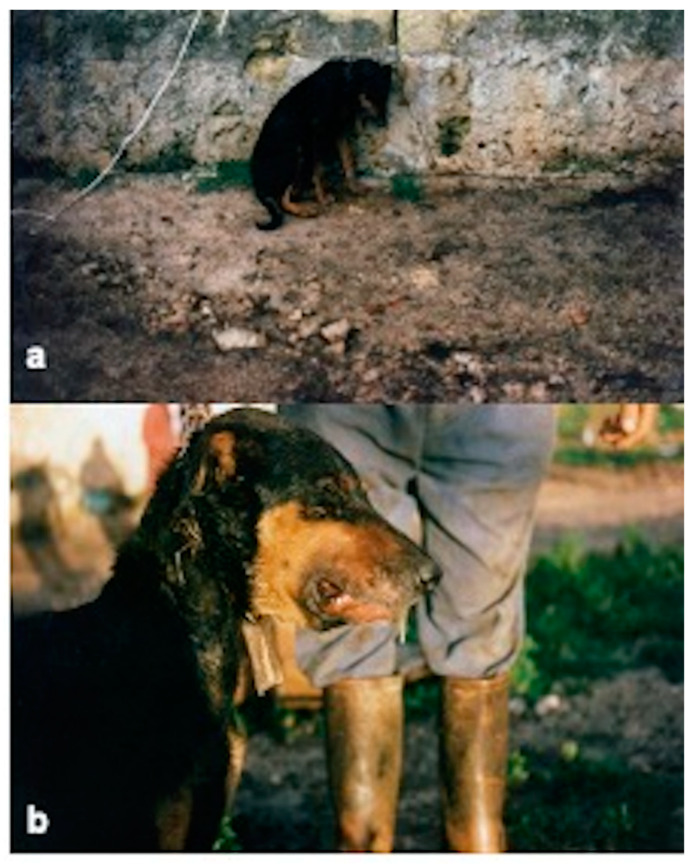
Dog with symptoms of Aujeszky’s disease. (**a**) severe prostration in the interval between one itch attack and another; (**b**) evident sialorrhea in the final stages.

The AD outbreak occurred in a mixed free-roaming farm of 145 sheep, 93 goats and 35 cattle near to a wild boar farm of 54 animals, located in the municipality of Giardinello in the province of Palermo (38.09581367263305, 13.157518721664935 Sicily-Southern Italy). The two farms can be considered as a single epidemiological unit, as the keepers of the animals are brothers and share the same grazing areas and the same watering sources. Therefore, in order to avoid repetitions, they will be described as a single farm. Cattle, sheep, and goats were reared in promiscuity and separated from the wild boar only by a metal fence. The farm did not have fences that could avoid contact with other farms or with wildlife. Both brothers were hunters, and one of them owned also a butcher’s shop. Often, hunted prey (wild pigs and wild boar) was dissected on the farm’s premises and the viscera were abandoned on the farmland. Furthermore, it was common practice to give slaughterhouse waste or processed meat to the dogs and cats on the farm and to feed the wild boar with kitchen scraps. Foxes searching for food were frequently observed in and around the farm. The farmers reported to the authorities the almost simultaneous (within 2–3 days) death of several animal species present on the farm. The official veterinary services of Palermo in collaboration with the diagnostic section of the Zooprophylactic Institute of Palermo, carried out the diagnostic investigations. In total 19 animals died in about a week, including 15 sheep, 2 goats, 2 cats and 2 dogs. Two foxes were also found dead (Figure 1) in an area close to the farms. Clinical symptoms included intense itching and neurological symptoms. The symptomatology and the proximity to the wild boar, led to the hypothesis of AD and as a precaution, animals were subjected to movement restrictions, except for handling intended animals to the slaughterhouse. An epidemiological investigation carried out in order to establish the possible source of the infection, revealed that about a month before (18/11/1996) the occurrence of the mortality cases (17/12/1997), 60 wild boar were purchased from a farm located in the municipality of Fara, province of Novara (45.562905, 8.458803; Piedmont—Northern Italy). The purchase was made to start a new wild boar farm. Before that, it was common practice to introduce boar, caught in the wild, to farms. The sale of the wild boar from north Italy was authorized by the veterinary services of Novara, after a clinical visit that had ascertained the good health of the animals. After the communication of the atypical high mortality by the veterinary services of Palermo (Sicily) to veterinary service of Novara (Piedmont—Northern Italy), diagnostic investigations were carried out in the wild boar farm of origin. Samples of serum and brain were taken from four randomly selected wild boar in order to verify the presence/absence of AD. The sera and brain samples were processed, respectively, for the detection of anti-gB and gE antibodies by ELISA tests and cultured by the virology laboratory of the Experimental Zooprophylactic Institute of Lombardy. Surprisingly, both tests were negative for AD, ascertaining the absence of the disease and excluding that the farm in Piedmont could potentially be the source of infection. The general clinical examination of symptomatic subjects still alive (a dog, a cat, a goat and a sheep), at time of inspection, showed non-specific symptoms such as anorexia, depression, restlessness, hyperthermia (39–40 °C), increased heart and respiratory rate. Moreover, in all species, severe itching was the most characteristic and constant symptom, presenting with hyperexcitability and continuous licking/scratching of different skin regions. While the dog and cat (Figure 2a) showed signs of itching in the facial region, in goat and sheep, the sites of itching were located in the posterior region of the body, particularly in the flanks and distal limbs. Severe self-inflicted traumatic alopecic lesions with haemorrhages, and crusts were observed in the itchy regions. In the final stages, aggressive behaviour and vocalization were evident in dogs and cats (Figure 2b), whereas sheep and goats showed rumen atony and tympanism. In all species an abundant salivary drain was present (Figure 3b), probably associated with the decrease of the swallowing reflex due to pharyngeal paralysis. Finally, the animals presented terminal recumbence and died within 2 or 3 days from onset of the first symptoms. No clinical symptoms were observed in cattle and the other species including wild boar. 

### 2.2. Serological Analysis

All 12 collected sera from wild boar were positive for the presence of anti-gE and anti-gB antibodies, confirming the circulation of SuAHV-1 in the wild boar population.

### 2.3. Necroscopy

In all the examined carcasses (three sheep, a goat, a cat, two dogs and two foxes), common pathological lesions were revealed, with some species differences (site of the itchy regions), in order to avoid repetitions, they are described together, underlining the differences in species. At the macroscopic examination the skin and skin appendages did not reveal any noteworthy alterations except for the presence of periocular and labial alopecic, hyperaemic and erosive lesions in the two dogs, the cat and the two foxes. Similar lesions were identified in the distal limbs and flank region of the sheep and the goat. The mucous membranes were hyperaemic and congested. Inspection of the thoracic and abdominal cavities and of the relative viscera revealed only minor lesions such as haemorrhagic spots in the spleen and liver. The brain and spinal cord, especially in sheep, showed, diffuse congestion, oedema and multifocal haemorrhagic foci affecting mainly the meninges and spinal cord.

### 2.4. Bacteriological and Virological Analyses

No bacteria were cultured from tissue samples of brain, spleen and liver using blood and Mac–Conkey agar plates. All the samples of brain examined for AD gave positive results at IFD confirming the presence of SuAHV-1 as the causative agent of AD in these animals. The biological test also gave positive results in the group inoculated with the nervous tissue samples. The rabbits developed clinical symptoms, including increased temperature, dyspnoea, stirring, restlessness, licking and biting the injection site on the neck, and finally, death, a few hours after the onset of symptoms.

## 3. Discussion

The present study reports a multi-species outbreak of AD that occurred in Sicily in 1996 involving, almost simultaneously, both domestic and wild species. The suspicion of AD was based on the farmer’s report (acute mortality preceded by neurological symptoms and pruritus) and the presence in the immediate proximity of wild boar, reared in the same area with which the different farmed species had continuous and both direct and indirect repeated contact. From the results of the clinical history and the epidemiological investigation, a cause-effect temporal correlation was hypothesized between the extra-regional purchase of wild boar and the onset of the disease in the Sicilian farm. The analyses carried out on the holding of origin (in Piedmont), however, gave negative results for AD, while the wild boar tested in the holding of destination by ELISA tests confirmed the circulation of the virus in the farm. Due to the lack of genetic and phylogenetic data on the strain involved in this study, it is possible only to hypothesize the mode of entry of AD in Sicilian farm. The simultaneous involvement of several animal species—both domestic (sheep, goats, dogs and cats) and wild (foxes)—with all different ethological behaviours, suggests a common source of infection. It is likely that SuAHV-1 was already circulating in the wild boar farm. In particular, the wild boar present in the farm (but caught in the wild) were already infected and the ones purchased from the north of Italy became infected upon arrival at the farm. To confirm this, analyses were conducted at the farm of origin which gave a negative result for SuAHV-1. Furthermore, from the follow-up data, the Sicilian wild boar farm continued to buy wild boar from the same farm after the outbreak was closed, and all the animals there purchased have always been negative for AD. Considering the high stability of Su-HV1 in the environment, and the total absence of a biosecurity measures in the farm, it is not unlikely that the environment was contaminated and that it served as a single source of infection for all the species [20]. On the farm, it was possible to observe the full extent of the sharing of spaces, feeding and watering areas between the different domestic species, as well as with wildlife. It was common practice to introduce wild pigs and wild boar, captured in a wild environment, to the farm for breeding purposes. Another common risky practice consists of dissecting the hunted wild boar on the farm and leaving their viscera on the ground, becoming a source of food for dogs, cats and foxes. Infection in non-natural hosts generally occurs by faecal-oral or aerosol routes, but, given the high stability of SuAHV-1 in the environment, indirect infection by exposure to infected fomites is also described [20]. Sheep and goats are mainly infected by aerogenous spread, but they are also highly susceptible to percutaneous infection [8]. Differently, both domestic (cats and dogs) and wild carnivores (foxes) can acquire the infection by the consumption of raw meat of infected pigs or wild boar [26,27], or by direct/indirect contact with infected animals [28,29] and/or carcasses. In our case, 15 sheep out of 145 and 2 goats out of 93 died in 2–3 days. Although goats are more susceptible to contagion and show more pronounced clinical manifestations, in sheep, morbidity can reach 60% [8]. This is likely due to the possibility of horizontal transmission [30,31]. The symptoms described in the present study were similar to those reported in the literature. The main and predominant symptom recorded in all the involved species is incessant itching. The itch is considered a typical sign of AD in species other than pigs and wild boar; it is typically classified as “neuropathic pruritus” [14]. Cases of AD without pruritus have been described in dogs [32] and horses [33]. Experimental studies on the pathogenesis of AD suggest that different localizations of pruritus are related to the pathway of the virus’ penetration. The head and neck are mainly involved when infection occurs via the oral and/or respiratory mucosa [14,34,35]. In case of virus exposure through the rectal and vaginal mucosa, pruritus typically develops in the shoulder, flank, hindlimbs and perineum [34]. In our case, in dogs and cats, the itchy areas were localized at the level of the face and neck, suggesting contagion by aerosol or oral mucosa, while, in the goat and sheep, they were localized at the flanks and the extremities of the hindlimbs, suggesting contagion via vaginal/rectal mucosa or following skin lesions. The macroscopic lesions observed during the necropsy are also similar to those reported in the literature [35,36,37,38]. Secondary traumatic lesions caused by intense itching are common in all species, as is congestion of the meningeal vessels and oedema [8]. Confirmation of AD was supported by the positive results for SuAHV-1 on IFD and biological test on nervous tissue samples, of a goat, three sheep, two foxes, a cat and a dog. This report has historical relevance, as it is the first documented outbreak of AD involving both domestic (sheep, goats, dogs and cats) and wild species (fox), as well as the first report of AD in goats, sheep and cats ever reported in Italy. In our country, the situation of AD in pigs has had, for many years, a variegated trend, with regions belonging to the three health qualifications referred to in Annex VI of the Implementing Regulation (EU) 2021/620. The province of Bolzano and the Friuli Venezia Giulia region have been listed in Part 1 of Annex VI of the 2021/620 Regulation as officially free from AD. In the last six years, considering that most European countries have achieved AD-free status, the regions of northern Italy with a prevalent swine vocation have taken steps to obtain a similar status in order to avoid significant economic sanctions. This led to the inclusion in Part 2 of Annex VI of regions with an approved eradication program for AD infection. Furthermore and recently, in April 2021, the rest of the national territory, except for the Region of Sardinia, obtained the approval of the eradication and control programs for AD by the European Commission and was included in Part 2 of the same annex. Despite progress in the control and elimination of AD in domestic pigs, there is continued evidence of infections in wild boar and hunting dogs, as reported by many studies conducted in Italy [2,3,39,40,41]). In addition, several other cases of AD were detected in non-natural hots such as foxes [15,16], wolf [17], captive brown bears [18], and cattle [19].

The recent outbreak reported by our research group in cattle in 2020 in Sicily [19] and the other cases of AD in dogs (unpublished data) suggest that AD, in Sicily, is widespread and has epizootic potential. Indeed, our research group diagnosed, in 2014 (unpublished data), two cases of AD in hunting dogs, likely due to the ingestion of infected wild boar meat, in the province of Messina (Sicily). The isolated strain showed the highest similarity with the SuAHV-1 strains isolated in wild boar and cattle [19], suggesting that there could be multiple sources of infection, along with interspecies virus transmission. These evidences suggest that AD in Sicily is and was prevalent, despite the measures applied to date. Moreover, the authors believe that AD in species other than pig and wild boar is strongly underestimated. This is probably due to the sporadic nature of the disease and the reporting of only striking cases. It is not uncommon to receive post-hoc informal reports of suspicion of AD in dogs used to hunt wild pigs and wild boar from freelance veterinarians. The absence of molecular epidemiology data does not allow us, to date, to make an analysis of the epizootic risk in Sicily. SuAHV-1 is typically a multi-host pathogen able to infect and cause disease in both domestic and wild animals [8] as well as humans [10,11,12,13]. The Sicilian zootechnical system is characterized by a prevalence of mixed farms (cattle, sheep, goats, pigs etc.), especially in protected natural areas where there are historically transhumant farms, where free-roaming animals share pastures and water sources with wildlife (foxes, martens, wild pigs, wild boar, etc.). In such a multi-host epidemiological context, it is clear that the epizootic risk increases, and that greater attention should be paid to management practices and the implementation of biosecurity measures. Although multiple human and financial resources have been focused on AD control, the disease is still prevalent in Sicily, with higher indices recorded in some protected natural areas [19]. The multi-species outbreak reported in this study occurred in December 1996, shortly before the application of the first obligatory national plan for the control and eradication of AD in Italy (DM. 11/04/1997). Until then there had only been a national voluntary plan for the eradication of the disease (D.M. 01/08/1994). Unlike other Italian regions, in Sicily, there had never been a regional control plan until 2020, therefore the national control plan has, since, been applied. In Italy, the current national control program of AD in pigs is based on serological surveillance, sanitary prophylaxis and vaccination (GURI—Decree 01/04/1997 Art.1). The use of gE-deleted live-attenuated vaccines for the breeding-pig category was allowed, experimentally, for two years (DDMM 30/12/2010 and 4/10/2011) and then authorized in 2013 for breeding pigs, but with maximum biosecurity conditions (Ministry of Health, circular of 17/05/2013). Twenty-three years from the entry in force of the national plan, the health department of the Sicily Region (Circular Health Department of the Sicily Region with protocol 00 21810 of 11 June 2020), introduced a regional plan that imposed more stringent control measures to reduce the spread of the virus in the Sicilian pig population. A mandatory management plan based on biosecurity, vaccination and the elimination of positive animals is required on each pig farm. Finally, in 2021, this region of Sicily was included in annex II of 2008/185/EC (version of 2021-03-04) regarding member states or regions, wherein approved national control program for the eradication of AD are in place. The Commission Delegated Regulation (EU) 2020/689 of 17 December 2019 supplementing Regulation (EU) 2016/429 of the European Parliament and of the Council as regards “rules for surveillance, eradication programmes, and disease-free status for certain listed and emerging diseases”, it also includes AD, and even stricter criteria are defined for assigning AD-free status to a member state or zone, including non-vaccination for at least 12 months and no clinical, virological and serological evidence of AD for 24 months (Part V, Chapter 2, Section 1, paragraph 1 points (a), (b), (c)). It is clear that, in light of the legislation in force and in view of the application of the measures for AD’s eradication, a timely analysis of the risk factors is required in Sicily. These data are essential to make effective the actions imposed by the enforced legislation. The main methods of controlling AD in the pig population include rigorous vaccination, based on the use of gE-deleted live-attenuated vaccines; serological diagnosis, using ELISA for the detection of specific gE antibodies to distinguish infected animals from vaccinated animals; quarantine; the elimination of positive animals, and above all, the application of strict biosecurity measures [42]. In light of the above and considering the increase in AD prevalence in Sicilian pig farms, especially in some areas [19], it is useful to underline that the currently enforced regional plan will be effective only if some critical issues that persist in some areas are addressed and resolved. In some areas of Sicily, such as protected natural areas and marginal areas, where pigs are typically reared in free-roaming zootechnical systems, applying adequate biosecurity measures is difficult. It is clear that for this type of farm, the use of the gE-deleted live attenuated vaccine must be prohibited, as clearly indicated by the Ministry of Health (Ministry of Health, circular of 17/05/2013), which recommends the “use only under conditions of ‘maximum levels of biosecurity’”. Vaccination plans must be rigorous, limiting those interventions on animals that can cause any form of stress at the time of vaccination (castration, antiparasitic treatments, branding) and that could compromise the effectiveness. The immediate elimination of positive subjects should also be encouraged. Finally, it is desirable that regional funding should be destined to the improvement of biosecurity measures (e.g., proper fences) in the farms and to proper training programs for freelance veterinarians and breeders. This article is historically relevant, not only because it reports the first cases of AD in goat, sheep and cat in Italy but because, if integrated with the recent acquisitions of AD in other species in multi-host contexts in Sicily, it suggests the spread of the disease and the potential epizootic risk. 

## 4. Materials and Methods

### 4.1. Anamnesis, Clinical and Epidemiological Investigation and Sampling

The staff of the official veterinary service of Palermo and the diagnostic section of the Zooprophylactic Institute of Palermo carried out several operational inspections, for diagnostic purposes, which involved both the farm with the ongoing outbreak and the wild boar farm close to it. During the inspections, both farms were subjected to clinical and epidemiological investigations and all the information was recorded and evaluated in the diagnostic process. In the farm where there was the ongoing infectious outbreak, clinical examination was performed on symptomatic animals present at the time of inspection, specifically on a dog, a cat a goat and a sheep. Evaluation of the basic clinical parameters (temperature, hydration status, heart and respiratory rate), inspection of the skin and evaluation of the neurological parameters were carried out. Remote clinical examination and recording of the anamnestic information from the farmer were pursued on the other asymptomatic species and animals present on site. The carcasses of two sheep, a goat, a cat, two foxes, and two dogs were collected and sent to the necroscopy service of the diagnostic section of the Zooprophilactic Institute of Palermo (Sicily) for pathological investigations. The neighbouring wild boar farm was also subjected to similar investigations. In particular, the anamnestic data were collected, a clinical examination was performed, and all lesions were recorded. In addition, blood samples (serum), were collected on 12 wild boar aged between 6–12 months, chosen as a sample out of the 54 present and sent to the virology laboratory of the Experimental Zooprophylactic Institute of Sicily.

### 4.2. Serological Analysis

Twelve serum samples were collected from wild boar and tested for the detection of antibodies against gB and gE antigens of SuAHV-1. The samples were processed in the virology laboratory of the section of the Zooprophylactic Institute of Palermo using an ELISA Kit (Enzyme Like Immunosorbent Assay) kit according to the standard laboratory procedures.

### 4.3. Necroscopy

The carcasses of three sheep, a goat, a cat, two foxes, and two dogs were subjected to necropsy in the diagnostic section of the Zooprophylactic Institute of Palermo. A careful pathological examination was performed on all the carcasses. All the lesions were recorded. Furthermore, during the necropsies, samples of brain (pons, brainstem, hippocampus, cerebellum), the thoracic and lumbosacral tracts of the spinal cord, liver and spleen were collected for bacteriological and virological investigations.

### 4.4. Bacteriological and Virological Analyses 

An aliquot of the brain samples, liver and spleen was used to perform aerobic and anaerobic bacterial cultures. Tissue samples were cultured using blood and Mac–Conkey agar plates. Another aliquot of the brain samples was used for virological investigations, such as cell culture, IFD, and a biological test on the rabbit. The tissue sample was inoculated into porcine kidney cell line PK-15; after incubation for 1 hour, the cells were washed and then cultured continuously until they produced an obvious cytopathic. The presence of the SuAHV-1 antigen was detected in the infected cell line as well as in brain tissues using immunoperoxidase with two MAbs specific to gB and gE proteins, respectively [43]. Two groups of eight rabbit were randomly selected for the biological test. The samples of nervous tissue were ground of each animal under sterile conditions and injected subcutaneously into the necks of eight 20-week-old male New Zealand white rabbits, another group of eight rabbits were instead inoculated with PBS in the same way as seen before. Both groups were kept under observation, any anomalies were recorded. 

## 5. Ethic Statement

All sampling procedures performed on live animals comply with good clinical practice and animal welfare. No ethical approval was required for this study, as no experimental procedures were pursued.

## Data Availability

Data sharing not applicable. No new data were created or analyzed in this study.
